# Evolution of ancient satellite DNAs in extant alligators and caimans (Crocodylia, Reptilia)

**DOI:** 10.1186/s12915-024-01847-8

**Published:** 2024-02-27

**Authors:** Vanessa C. Sales-Oliveira, Rodrigo Zeni dos Santos, Caio Augusto Gomes Goes, Rodrigo Milan Calegari, Manuel A. Garrido-Ramos, Marie Altmanová, Tariq Ezaz, Thomas Liehr, Fabio Porto-Foresti, Ricardo Utsunomia, Marcelo B. Cioffi

**Affiliations:** 1 Departamento de Genética E Evolução, Universidade Federal de São Carlos, São Carlos, São Paulo, Brazil; 2 Faculdade de Ciências, UNESP, Bauru, São Paulo, Brazil; 3 Departamento de Genética, Facultad de Ciencias, Universidad de Granada, 18071 Granada, Spain; 4 Institute of Animal Physiology and Genetics, Czech Academy of Sciences, 27721 Liběchov, Czech Republic; 5 Department of Ecology, Faculty of Science, Charles University, 12844 Prague, Czech Republic; 6 Institute for Applied Ecology, University of Canberra, Canberra, Australia; 7 Institute of Human Genetics, Jena University Hospital, Friedrich Schiller University, Jena, Germany

**Keywords:** Repetitive DNA, Reptiles, Library hypothesis

## Abstract

**Background:**

Crocodilians are one of the oldest extant vertebrate lineages, exhibiting a combination of evolutionary success and morphological resilience that has persisted throughout the history of life on Earth. This ability to endure over such a long geological time span is of great evolutionary importance. Here, we have utilized the combination of genomic and chromosomal data to identify and compare the full catalogs of satellite DNA families (satDNAs, i.e., the satellitomes) of 5 out of the 8 extant Alligatoridae species. As crocodilian genomes reveal ancestral patterns of evolution, by employing this multispecies data collection, we can investigate and assess how satDNA families evolve over time.

**Results:**

Alligators and caimans displayed a small number of satDNA families, ranging from 3 to 13 satDNAs in *A. sinensis* and *C. latirostris*, respectively. Together with little variation both within and between species it highlighted long-term conservation of satDNA elements throughout evolution. Furthermore, we traced the origin of the ancestral forms of all satDNAs belonging to the common ancestor of Caimaninae and Alligatorinae. Fluorescence in situ experiments showed distinct hybridization patterns for identical orthologous satDNAs, indicating their dynamic genomic placement.

**Conclusions:**

Alligators and caimans possess one of the smallest satDNA libraries ever reported, comprising only four sets of satDNAs that are shared by all species. Besides, our findings indicated limited intraspecific variation in satellite DNA, suggesting that the majority of new satellite sequences likely evolved from pre-existing ones.

**Supplementary Information:**

The online version contains supplementary material available at 10.1186/s12915-024-01847-8.

## Background

Eukaryotic genomes are highly variable in structure and size because of the presence of vast quantities of repetitive DNA [1, 2]. Satellite DNAs (satDNAs) are a common component, accounting for an important part of the genome in most animal and plant genomes (reviewed by [3–5]. In general, a genome has a varied number of satDNA families (the satellitome) [6] with varying nucleotide sequences and genomic abundance [7–13]. Although some examples of small arrays scattered throughout euchromatin have been documented [6, 14–22], these sequences are often found in centromeres and in pericentromeric and subtelomeric heterochromatic areas [4, 10, 23]. More than just “junk DNA” (as for a long time considered), several studies have revealed that satDNAs have a role in a variety of biological processes, including gene regulation [24], centromere function [25], chromatin modulation [26], and spatial chromosomal structure [27–29]. The combination of cytogenetics and genomics studies has proven to be useful in elucidating numerous aspects of genome evolution and organization [30, 31], with particular emphasis on repetitive DNAs [6, 32–34]. Furthermore, due to their tandemly repeated genomic organization, satDNA studies in non-model organisms were boosted in the last few years, especially with the development of several assembly-free pipelines designed for using raw reads [35–38]. In this context, several satDNA catalogs were characterized in a variety of invertebrate and vertebrate species [6, 34, 39–44].

Although related species sharing a common ancestor share the same ancestral library of satDNA families, differential amplification of the different satDNAs, and different variants of each of them, leads to differentiated satDNA profiles in each species [5, 7, 10]. This involves the replacement of some satDNA families by others at specific sites on chromosomes such as centromeres, for example. The rate of change is very rapid, and satDNA sequences represent one of the fastest evolving genomic components. This often leads to high levels of interspecific sequence diversity even within closely related species, exhibiting very different profiles (both quantitative and qualitative) of satDNAs in their genomes [4, 10, 45]. However, satDNA rate of change can be altered (accelerated or slowed) by various factors such as the location and organization of the repeated sequences [46], functional constraints [42, 47–51], biological factors [8, 11], or population and evolutionary factors [41, 52, 53]. In this regard, it is particularly intriguing to investigate why, for some satDNAs, this process is slower than expected and persists over long periods, spanning dozens (or even hundreds) of million years, in the same chromosomal location in the entire group of related species [32, 42, 47, 52–56]. It is also important to examine the effects of slow rates of molecular and morphological evolution described in a species group [52] on the evolution of the satellitome.

Crocodilians are one of the oldest extant vertebrate lineages, demonstrating evolutionary success and morphological resilience over many millions of years [57]. Extant species have preserved physical and ecological traits for nearly 100 million years, unlike other vertebrates that have undergone significant diversity [58–60]. Crocodilians have a key position in vertebrate phylogeny because, combined with dinosaurs, pterosaurs, and modern birds, they compose the archosaurs, a monophyletic group [61–63]. Crocodylia is classified into three families: Crocodylidae, Gavialidae, and Alligatoridae, with approximately 27 species [64]. The family Alligatoridae is made up of eight species that are divided in four genera: *Melanosuchus, Paleosuchus*, and *Caiman*, which belong to the Caimaninae subfamily and *Alligator*, which forms the monogeneric subfamily Alligatorinae. Except for the *Alligator* genus, where *A. mississippiensis* and *A. sinensis* are limited to the Southeastern United States and China, respectively, all the other six species are presently found in South America, being more widespread in Brazil [57, 65].

The karyotypes of all current Alligatoridae species were recently revised using conventional differential staining and up-to-date molecular cytogenetic approaches [66]. Although there is a limited amount of diversity and a certain level of karyotype stasis (with diploid numbers equal to 2n = 42 and 2n = 32 for all Caimaninae (*Caiman, Paleosuchus,* and *Melanosuchus*) and Alligatorinae (*Alligator)* species, respectively), their genomic content revealed significant interspecific divergence [66].

Here, we performed the first broadscale comparative analysis of the alligators’ satellitomes. By combining genomic and chromosomal data, we identified and compared the full catalogs of satDNA families (i.e., the satellitomes) of 5 of the 8 extant Alligatoridae species, revealing ancestral patterns of evolution and enabling investigation into how satDNA families evolve over time. The results revealed strong sequence conservatism among Alligatoridae species with very limited diversity of their satDNA library. Furthermore, fluorescence in situ assays in all the 8 extant Alligatoridae species showed that the identical satDNA orthologs can exhibit various hybridization patterns, indicating their high evolutionary dynamics.

## Results

### Bioinformatic satDNA characterization

After several iterations (*C*. *yacare* = 5, *C*. *latirostris* = 7, *M*. *niger* = 4,* P*. *trigonatus* = 4, and *A*. *sinensis* = 2), we characterized 39 satDNAs in Alligatoridae, where repeat unit lengths ranged from 23 (PtrSat11-23) to 6317nt (ClaSat02-6317) and the average of their A + T content was 46.9%. Specific features of the satDNAs in each species are summarized in Table 1. The number of iterations performed for each species was a consequence of the results obtained in each round so that when no new tandem repeats were discovered in a given round the analysis was not continued. Thus, for example, in the case of *A. sinensis* no tandem sequences were discovered in the third interaction. Iterations using RepeatExplorer2 after TAREAN did not return any characterized satellite DNA for the five species analyzed.

**Table 1:** General features of Alligatoridae satellitomes characterized with TAREAN. SF = superfamily, RUL = repeat unit length, TSI = tandem structure index. Divergence per satDNA was expressed as the percentage of Kimura divergence. SatDNAs that have 50% or more identity belong to the same group

**Species**	**SF**	**satDNA**	**RUL**	**A + T (%)**	**Abundance (%)**	**Divergence (%)**	**TSI**	**Group**
*Caiman yacare*	1	CyaSat01-41	41	63.4	2.211	8.79	0.91	1
		CyaSat02-40	40	50.0	0.212	8.35	0.98	3
	2	CyaSat03-60	60	41.7	0.099	18.50	0.98	2
	1	CyaSat04-41	41	58.5	0.084	21.64	0.89	1
	2	CyaSat05-200	200	43.5	0.062	8.67	0.96	2
	1	CyaSat06-286	286	57.3	0.061	4.29	0.61	1
*Caiman latirostris*	1	ClaSat01-41	41	46.3	1.077	11.70	0.93	1
		ClaSat02-6317	6317	51.0	0.733	7.94	0.87	
	2	ClaSat03-183	183	40.4	0.268	9.30	0.87	2
		ClaSat04-536	536	26.1	0.262	4.83	0.92	
		ClaSat05-40	40	47.5	0.207	9.51	0.87	3
	1	ClaSat06-1063	1063	53.3	0.181	9.09	0.74	1
	1	ClaSat07-320	320	53.1	0.129	8.40	0.89	1
	1	ClaSat08-800	800	55.9	0.129	14.84	0.99	1
	1	ClaSat09-285	285	46.5	0.074	3.90	0.95	1
	2	ClaSat10-60	60	40	0.069	17.79	0.4	2
	1	ClaSat11-547	547	51.7	0.069	9.81	0.69	1
	2	ClaSat12-24	24	33.3	0.053	16.21	0.82	2
		ClaSat13-398	398	32.7	0.042	8.61	0.41	
*Melanosuchus niger*	1	MniSat01-41	41	58.5	1.582	8.99	0.87	1
	1	MniSat02-246	246	55.7	0.442	5.54	0.74	1
		MniSat03-60	60	40.0	0.240	17.03	0.96	2
		MniSat04-40	40	52.5	0.110	11.23	0.97	3
	1	MniSat05-248	248	55.6	0.045	10.56	0.57	1
	1	MniSat06-41	41	56.1	0.032	18.32	0.90	1
*Paleosuchus trigonatus*	1	PtrSat01-41	41	41.5	1.092	05.02	0.91	1
	2	PtrSat02-24	24	37.5	0.705	10.34	0.98	2
	1	PtrSat03-147	147	55.1	0.594	10.02	0.95	1
	1	PtrSat04-99	99	51.5	0.332	04.07	0.72	1
	1	PtrSat05-41	41	53.7	0.328	12.86	0.92	1
	2	PtrSat06-100	100	45	0.252	9.69	0.97	2
	1	PtrSat07-41	41	56.1	0.155	16.09	0.96	1
	3	PtrSat08-214	214	28	0.14	01.05	0.57	3
		PtrSat09-490	490	31.6	0.112	4.29	0.96	
	3	PtrSat10-40	40	45	0.112	12.41	0.38	3
	1	PtrSat11-23	23	52.2	0.028	13.70	0.05	1
*Alligator sinensis*		AsiSat01-1717	1717	50.6	0.234	13.39	0.95	
		AsiSat02-60	60	38.3	0.154	3.51	0.99	
		AsiSat03-96	96	34.4	0.02	9.25	0.93	3

In general, alligators and caimans analyzed here exhibited few satDNAs (minimum of three in A. sinensis and maximum of 13 in *C. latirostris*), and also a small diversity in the within- and between-species level. Intraspecific cases with similarity greater than 50% and less than 80% were classified as the same superfamily, while interspecific cases with similarity greater than 50% were placed in the same group, which was further subdivided into four distinct ones.

Based on sequence alignments, four main groups of satDNAs were identified showing at least 50% of similarity that encompassed satDNAs from at least four species, named here as group 1 (*N* = 19 satDNAs shared among Caimaninae), group 2 (*N* = 8 satDNAs shared among Caimaninae), group 3 (*N* = 6 satDNAs shared among Alligatoridae), and group 4, with satDNAs from two species (*N* = 2 satDNAs shared among Caimaninae) (Additional file 1: Fig. S1; Additional file 2: Table S1; Table 1), the remaining 4 satDNAs did not show any similarity with other sequences (Table 1). This classification helped us to delimit the origin of some satDNAs and follow their diversification patterns in each species.

**Fig. 1:**
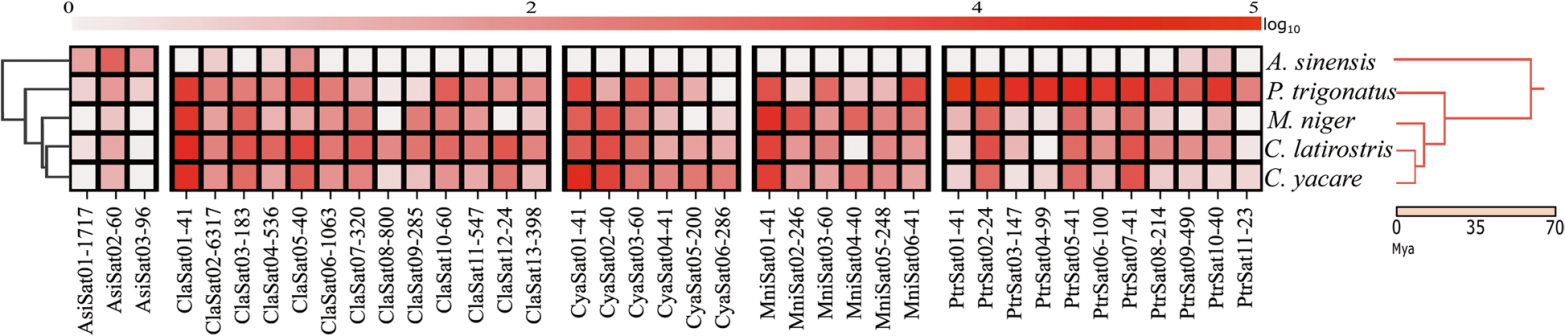
Clustermap evidencing presence/absence and abundance of the satDNAs across Alligatoridae species. Abundances were calculated as the log10 of the proportion of short reads masked as satDNAs with a maximum of 20% of divergence and normalized by single-copy genes. On the left, hierarchical calculated clusters; on the right, species name and the proposed phylogeny for the group with their respective divergence times, based on data generated by [65]

We also performed one comparative RepeatExplorer2 run, inputting reads from all the analyzed species into a single dataset. Results obtained corroborated our previous analyses and satDNAs from groups 1–4 were found, as well as other tandem repeats not classified within these groups (Additional file 2: Table S2). A general clustermap considering the genomic abundances of satDNAs exhibiting a maximum of 20% of divergence in each species was also generated and is in accordance with the phylogenetic relationships (Fig. 1). As expected by theory, the more distant two species are, the fewer satDNAs they share. For instance, *A. sinensis* almost does not share satDNAs with the other species.

**Fig. 2:**
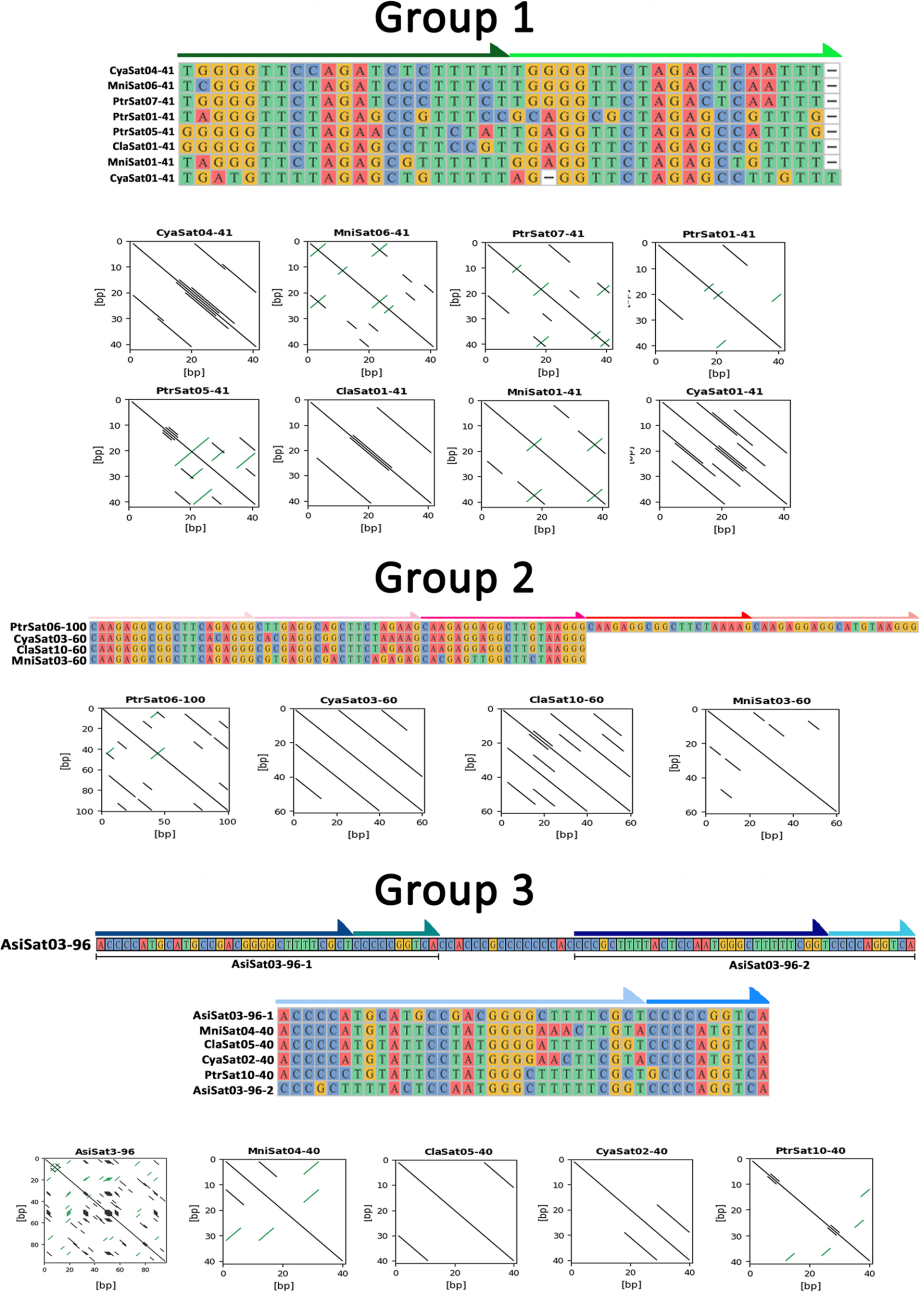
Sequence alignment of satellite repeats in groups 1, 2, and 3, demarcated by arrows in the alignments, representing the subunits from which the sequences in each group originated. In addition, dotplots of each satDNA, presenting the internal repetitions among all monomers, are also indicated

Expanding our analyses, as we found a significant RUL variability in each of those groups, we generated a global dot-plot with sequences from the abovementioned first three groups (groups 1, 2, and 3) (Fig. 2). As expected, sequences belonging to a same group showed similarities as revealed by the dotplots, which also indicated that most longer satDNAs within groups probably emerged from the diversification of pre-existing shorter satDNAs.

We generated dotplots for each satDNA monomer supporting this view (Fig. 2). Thus, for example, among group 1 satDNAs we observed that the 41-bp satellites are made up of a structure composed of two subrepeats (21 + 20 bp) (Fig. 2). Comparisons between these two subrepeats suggest that a satDNA of about 20 bp in length must have existed and that a new satDNA composed of 41-bp repeats emerged through a process of duplication and subsequent divergence (in fact, one of the satellites in this group, PtrSat11-23, is 23 bp long). Therefore, when comparing the mean divergence that exists between 41-bp repeat units (inter-repeat divergence) with the mean divergence that exists between 20/21 bp subunits that compose each repeat (intra-repeat divergence), we always find that the former is smaller than the latter (Additional file 2: Table S3 and Additional file 1: Fig. S2). Satellites of this group with lengths longer than 41 bp show a complex pattern of several cycles of duplication and divergence of subunits of about 40 bp or more. For example, the analysis of ClaSat06-1063 demonstrates a complex evolutionary pattern based on different cycles of duplication and divergence of sub-repeats of approximately 40/80 bp (including intervening sequences) (Additional file 1: Fig. S3). Similarly, group 2 satellites that are composed of 60 bp repeat units have a pattern of 20 bp subunits that again point to a formation of 60-bp satellites from smaller satellites (in fact, one of the satellites in this group, ClaSat12-24, is 24 bp long) (Fig. 2). Also in this case, mean inter-repeat divergence is smaller than mean intra-repeat divergence (Additional file 2: Table S4). Finally, group 3 is the only example of shared satDNAs between all the analyzed species here. Repeat monomers of 40 bp are predominant among these satDNAs, and dot-plot analysis revealed a heterogeneous structure based on two different subrepeats (29 bp + 11 bp, Fig. 2). In this case again, we show that mean intra-repeat divergence is greater than mean inter-repeat divergence (Additional file 2: Table S5 and Additional file 1: Fig. S4). Remarkably, in *A. sinensis*, a 96-bp-long satDNA was characterized in this group, and its monomer sequence reveal a complex structure composed of two 40-bp subrepeats and an intervening sequence (40 bp + 16 bp + 40 bp, Fig. 2).

BLAST searches against the genome of *A. sinensis* revealed that satDNAs classified as groups 1 and 2 were not found in this species, while matches were observed for sequences belonging to group 3 (CyaSat02-40, ClaSat05-40, MniSat04-40, PtrSta10-40, and AsiSat03-96) and group 4 (ClaSat04-536 and PtrSat09-490). BLAST searches also resulted in matches against ClaSat02-6317 and ClaSat13-398, satDNAs that are not classified in any group (results are summarized in Additional file 2: Table S6). These results suggest that groups 1 and 2 of sequences emerged after the split of Caimaninae and Alligatorinae, while groups 3 and 4 are shared among the representatives of both subfamilies. In addition, Alligatorinae-specific AsiSat01-1717 and AsiSat02-60 satDNAs returned abundant significant matches, as expected.

ClaSat02-6317 and ClaSat13-398 produced multiple hits against the *A. sinensis* genome (*n* = 6264 and 1712, respectively). Remarkably, the obtained TSI for ClaSat13-398 was low (TSI = 0.21), suggesting that this sequence is dispersed along the genome. While ClaSat02-6317 exhibited a higher TSI (TSI = 0.74), we hypothesize that this is due to its larger monomer size. Since the fragments of paired-end sequencing are usually around 300–400 bp and the monomer of this satDNA is > 6000 bp, the obtained TSI is most likely due to mapping in the same monomer, not mapping in adjacent monomers. In fact, both ClaSat02-6317 and ClaSat13-398 do not show FISH hybridization signals in this species supporting their scattering as short tandems throughout the genome. Interestingly, a RepeatMasker search on the vertebrate database of Repbase revealed that the former is homologous to endogenous retroviruses (ERVs) (62% identity; 70% of the element) and the latter to LINE sequences (70% identity; 53% of the element) (Supplemental Table S7). On the other hand, this search also revealed that AsiSat1-1717 shared a 54 and 82% of its sequence with two satellite DNAs previously found in the Nile crocodile (NCBI accession numbers: OP480175 and OP480176).

### Chromosomal location of satDNAs with differential abundance between species

We analyzed the chromosomal location of satDNAs that were successfully amplified by PCR belonging to group 1 (ClaSat01-41; ClaSat06-1063; ClaSat07-320; ClaSat08-800 and ClaSat11-547), group 2 (ClaSat10-60), group 3 (ClaSat05-40), and group 4 (ClaSat04-536) in addition to the two exclusively ones found in *A. sinensis* (AsiSat01-1717 and AsiSat02-60) in all Alligatoridae species to check their chromosomal distribution. Additionally, the ungrouped satellites ClaSat02-6317 and ClaSat13-398 were tested but none of them yielded visible FISH signals in any species (data not shown).

**Fig. 3:**
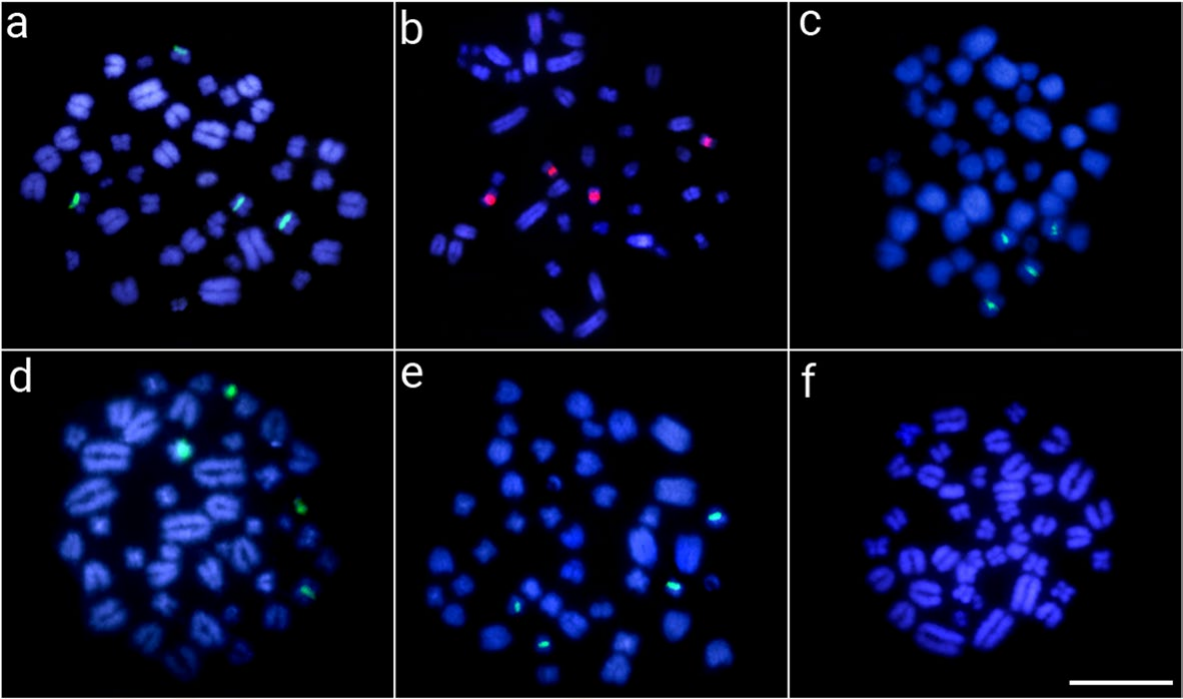
Metaphase chromosomes from *C. crocodilus* (**a**), *C. latirostris* (**b**), *C. yacare* (**c**), *M. niger* (**d**), *P. palpebrosus* (**e**), and *P. trigonatus* (**f**) after in situ mapping of ClaSat01-41 (group 1). The satDNA FISH signals are highlighted in green (ATTO488 labeled) or red (ATTO550 labeled) and the chromosomes were counterstained with DAPI (blue). Scale bar = 20 μm

**Fig. 4:**
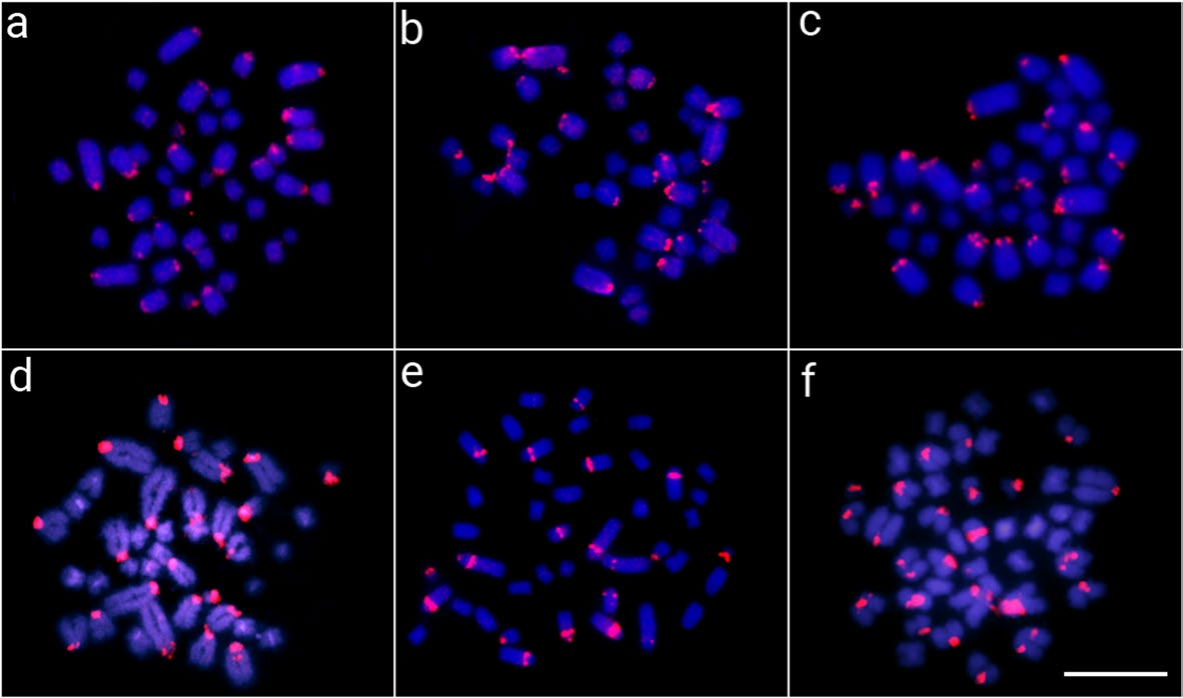
Metaphase chromosomes from *C. crocodilus* (**a**), *C. latirostris* (**b**), *C. yacare* (**c**), *M. niger* (**d**), *P. palpebrosus* (**e**), and *P. trigonatus* (**f**) after in situ mapping of ClaSat10-60 (group 2). The satDNA FISH signals are highlighted in red (ATTO550 labeled) and the chromosomes were counterstained with DAPI (blue). Scale bar = 20 μm

**Fig. 5:**
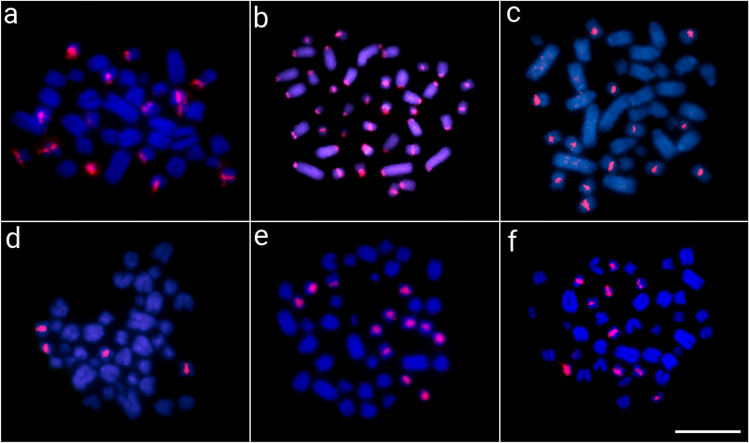
Metaphase chromosomes from *C. crocodilus* (**a**), *C. latirostris* (**b**), *C. yacare* (**c**), *M. niger* (**d**), *P. palpebrosus* (**e**), and *P. trigonatus* (**f**) after in situ mapping of ClaSat05-40 (group 3). The satDNA FISH signals are highlighted in red (ATTO550 labeled) and the chromosomes were counterstained with DAPI (blue). Scale bar = 20 μm

Concerning the ClatSatDNAs, except for the satellite ClaSat04-536 (group 4), which showed no hybridization signals in any species, all the other satDNA sequences were found in (peri-) centromeric heterochromatin regions in all Caimaninae species **(**Figs. 3, 4, and 5). Both alligators (*A. sinensis* and *A. mississippiensis*) showed no hybridization signal for any of the ClaSatDNAs investigated (data not shown), which is in accordance with the clustermap analysis. Here, to illustrate, we present the results for representative selected ClaSatDNAs from each of the major groups identified (Figs. 3, 4, and 5). The satDNA ClaSat01-41, belonging to group 1 (the most frequent group present in each species), were mapped in two chromosomal pairs in all Caimaninae species except *P. trigonatus*, which did not display any hybridization signal (Fig. 3). However, despite sharing the same motifs, some divergent and species-specific chromosomal location patterns were observed among ClaSatDNAs from group 1 among species (Additional file 1: Figs. S5–S6). For satellites in groups 2 and 3, numerous chromosomal pairs containing these sequences were found in nearly all species (Figs. 4 and 5). *M. niger* is distinctive for displaying hybridization signals on only two chromosomal pairs for group 3 satellites (Fig. 5d).

**Fig. 6:**
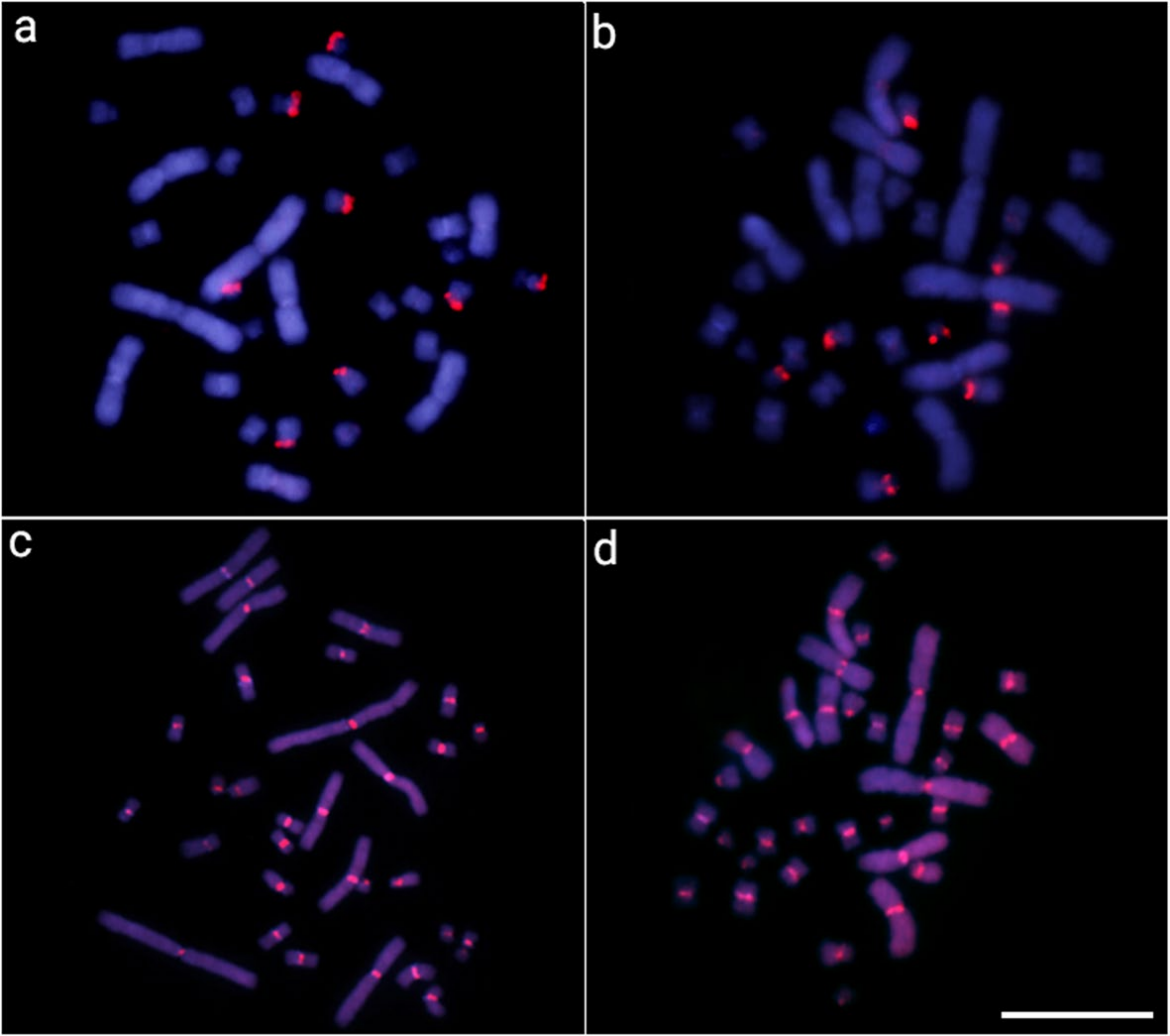
Metaphase chromosomes from *A. sinensis* (**a** and **c**) and *A. mississippiensis* (**b** and **d**) after in situ mapping with AsiSat01-1717 (**a** and **b**) and AsiSat02-60 (**c** and **d**) probes. The satDNA FISH signals are highlighted in red (ATTO550 labeled) and the chromosomes were counterstained with DAPI (blue). Scale bar = 20 μm

Besides, we also mapped the two exclusive satDNAs presented in *A. sinensis* genome (AsiSat01-1717 and AsiSat02-60) in all Alligatoridae species. Both AsiSatDNAs showed hybridization signals only in *Alligator* species. While AsiSat01-1717 was exclusively mapped in several chromosomes, AsiSat02-60 was mapped in all centromeres of both species (Fig. 6). Collectively, our analyses revealed, for *A. sinensis*, that (i) although group 3 and group 4 satellites are present in the A. sinensis genome, these satellites are poorly represented and possibly organized in short tandems scattered throughout the genome as can be deduced from TSI values, BLAST search and FISH: hybridization signals were not visible, and satellites exhibited high TSI, but low number of alignments in BLAST; and (ii) this is in contrast to the alligator-specific satellites that appear clustered at loci on long arrays, consistent with results obtained in FISH experiments in which these satellites give conspicuous FISH bands, high TSI values, and a large number of alignments in BLAST.

## Discussion

Despite the fact that both alligators’ (*A. sinensis* and *A.mississippiensis*) complete genomes were characterized some years ago [63, 67, 68], genome-wide investigations of satDNAs in this group of organisms were never undertaken. SatDNAs are well known to be underrepresented in genome assemblies [4], particularly those genomes assembled using short-read sequencing technology, as is the case with alligators. In this context, knowledge about satDNAs in crocodilians was limited to just a few works [69, 70]. Given that high-throughput satellitome analysis has been very enlightening for understanding the satDNA evolution in various organisms, we used a chromosome- and genomic-based approach to try to describe the satellitome from members of all current Alligatoridae genera for the first time. In a period of around ~ 70 Myr (million years), many satDNA sequences are shared among the species, assisting in the hypothesis that they are derived from small sequences, as shown in Fig. 2. Furthermore, in following fluorescence in situ tests the distinct hybridization patterns for the identical ortholog satDNAs were observed.

After mining satellite DNAs using well-established bioinformatic pipelines [6, 36], we found that alligators’ satellitomes are among the smallest catalogs described until now, varying between 3 and 13 satDNAs, in *A. sinensis* and *C. latirostris*, respectively. In recent years, several satellitomes from a wide range of species, including plants and animals, have been identified [6, 42–44, 71–73]. These investigations showed that satellite DNA profiles are very dynamic. For example, characiform fish satellitomes display a significant quantitative and qualitative variation, with some species exhibiting a few dozen [44], while others can show more than one hundred satDNAs [74]. Here, we found that all alligators are similarly satDNA-poor constituting a common trend in this group.

Novel satDNA families can emerge by variable mechanisms and from multiple genomic regions, like introns, transposable elements, and/or existing satDNA families [38, 75, 76]. Our findings indicated that there was little intraspecific variation in satellite DNA, indicating that most new satellite sequences evolved from pre-existing ones. For instance, *C. latirostris* exhibited 13 satDNAs, but six and three of them were grouped as superfamilies (sequences showing more than 50% of similarity and less than 80%), named here as groups 1 and 2, respectively (Table 1). Interestingly, this limited diversity is also apparent at the interspecific level, where over 90% of the 39 satDNAs described for Alligatoridae can be categorized into 4 main groups of sequences. After their origin, new longer satellites derived from the complex diversification of shorter ones already existing in the genome throughout different and successive cycles of duplication and divergence, which has been extensively documented in other species [46, 52, 72].

The long-term evolution of satellite DNA catalogs in related species can be explained by the library hypothesis. Fundamentally, it states that changes in the profiles of satDNAs among species are mostly quantitative in the “library,” rather than multiple de novo origins [77]. Here, we could track the origin of the ancestral forms of satDNAs belonging to groups 1–4 to, at least, the common ancestor of Caimaninae (groups 1, 2, and 4) and Alligatoridae (group 3). We observed a substantial degree of similarity in satDNAs among species, with only four being species-specific. The long-term maintenance of satDNAs is notable. In this context, the conservation could be related to the acquisition of cellular function [42, 47–51, 77], particular genomic organization [32], or slow rates of evolution [52]. Previous studies found slow rates of molecular evolution within crocodilians [63]; thus, we hypothesize that satDNAs also evolved slowly in this group (as discussed below). In squamate reptiles, while the great majority of sequences are of recent origin and only observed in closely related species [78–83], several (and most common ones) are largely conserved in unrelated species [84].

The chromosomal mapping analysis revealed that all characterized satellites showed the general same chromosomal location (i.e., large peri- and centromeric blocks) among species, showing specific patterns for each one (Figs. 3, 4, 5, and 6 and Additional file 1: Figs. S5–S6). On the other hand, it is interesting to see that group 1 satellites, even being the most abundant in the Caimaninae genome, show a visible block of FISH signal in only two chromosomal pairs. When using the FISH technique, as a specific satDNA sequence can actually display a variety of array structures (dispersed and/or clustered into long and nonrandom arrangements) among species, it results in a range of labeling patterns at the chromosomal level. This is particularly true, for example, for the group 3 ClatSat05-40 because, although being abundant in the genome of *A. sinensis* (as indicated by our BLAST results in Additional file 2: Table S6 and clustermap using RepeatMasker data), it exhibits a non-cluster organization, which hindered in situ experiments from producing any detectable hybridization signals at the chromosomal level. We hypothesize that this could well explain the FISH patterns observed in Caimaninae for group 1 satDNAs, although we cannot verify this as we do not have the complete sequence of their genomes nor are these satDNAs present in the *A. sinensis* genome for comparisons. In this context, different satellites of groups 1 and 2 show TSI values that are compatible with a dual organization, both forming loci visible by FISH and forming short arrays scattered throughout the genome not detectable by FISH (Table 1).

On the other hand, it is remarkable that two of the satellites studied in this paper (ClaSat02-6317 and ClaSat13-398), which appear to be dispersed according to BLAST and FISH results, are related to mobile elements and show homology of an important part of their sequences with such elements, which suggests that these satellites have evolved from this type of elements. Specifically, ClaSat02-6317 is related to ERVs, while ClaSat13-398 is related to LINEs. There is increasing evidence that TEs are a major source of satellites (Šatović-Vukšić and Plohl, 2023) and these results support this proposal. Interestingly, it has been shown that the majority of within-crocodilian TE activity is derived from ERVs (Chong et al. 2014; Sotero-Caio et al. 2017). Our results therefore also support that these elements can constitute a source for satellites in Crocrodylia.

Our current findings are in line with the karyotype patterns described for the family, which show a stable dichotomy between the genera *Alligator* (2n = 32) and *Caiman, Melanosuchus,* and *Paleosuchus* (2n = 42), with 2n = 32 representing the most likely ancestral state [revised in 66]. The two main divergent karyotype groups to which these reptiles belong are reflected in both the specificities of their respective satDNA libraries in terms of their sequence composition and chromosomal locations. However, all the satDNAs were mapped in the constitutive heterochromatin that is limited to the pericentromeric areas in all Alligatoridae species [66]. It is reasonable to consider that some of these satellites would be a component of the centromeric chromatin, much like in other species [4, 5]. Although the presence of multiple dispersed loci composed of a single copy or a few tandem copies of a satDNA is a fact today [23], the accumulation of satDNAs (as well as other repetitive DNA families) in centromeres and in heterochromatic regions is characteristic, as observed in many other groups [4, 23, 85–87]. Such colocalization (i.e., the tendency to occupy similar locations on non-homologous chromosomes) might have been facilitated by the reunion of centromeres at the first meiotic prophase bouquet [6, 88]. This is especially true in Caimaninae since the karyotypes of all species are dominated by acrocentric chromosomes. In this context, the existence of large and small chromosomes in Caimaninae could be favoring the structural differences at the (peri)centromeric level between different chromosomes [89].

Both alligator species, *A. sinensis* and *A. mississippiensis*, displayed hybridization signals only for two (AsiSat01-1717 and AsiSat02-60) among all the investigated satDNAs (Fig. 6). Furthermore, AsiSat02-60 was exclusively mapped in all centromeres of both *Alligator* species. That is, these two species have conserved the same (peri)centromeric satDNA in all their chromosomes underscoring its possible important role in the centromeric and pericentromeric organization, a role that it may be shared with AsiSat01-1717 in some chromosomes. Alligatorinae long diverged (~ 70 Myr) from all the other Caimaninae and have highly rearranged karyotypes (2n = 32) that are predominantly metacentric, in contrast with all Caimaninae species that have 2n = 42 chromosomes and karyotypes dominated by acrocentric chromosomes [66]. We have proposed that 2n = 32 represents the likely ancestral state and that the karyotype diversification in Caimaninae was followed by a series of Robertsonian rearrangements in which centric fissions played a key role [66]. Accordingly, alligators’ satellitomes are among the smallest catalogs described until now for any species, with only 3 satDNAs identified.

Taking together the data obtained in this work, we can conclude that this group of ancient species that have survived on Earth for more than 100 Myr, has a very small common catalog of satDNA families. Nevertheless, each of the two lineages analyzed (Caimaninae and Alligatorinae), which have diverged for more than 70 Myr, is differentiated by the satDNAs that have been amplified in each group at the centromeric level. What stands out in this study is that these satellites have been conserved during all this time and persist for reasons that we have to analyze below. While the same satellite has been conserved in centromeres of Alligatorinae species for about 70 Myr, the chromosomal rearrangements that have taken place in the Caimaninae lineage would have caused the emergence and diversification of new satellite DNAs that have replaced them in the (peri)centromeric regions. Some of them, such as those of Group 3, were already present in a dispersed form in the ancestral genome of Alligatoridae, as was possibly the case with the satDNAs of Group 4 and the ungrouped satDNAs ClaSat02-6317 and ClaSat13-398 (Additional file 2: Table S2), still dispersed in all Alligatoridae species. In fact, the replacement of some satDNAs by others is common at the centromeric level even among closely related species in both animals and plants (reviewed in [4, 5, 90], see also the “Background” section). In the case of Alligatoridae, the slow evolution of their genomes may also be affecting in turn, as it was suggested for satDNAs from sturgeons [52, 53]. Extant crocodiles have limited rates of morphological [91, 92], molecular [63], and karyotype diversification [66, 93, 94]. Likewise, the present-day satellitome (particularly the Caimaninae species) shares common satDNA libraries among its species, despite their long time of divergence. Therefore, the following questions arise: (a) why have they also changed so little in such a highly variable genome fraction over such an enormous span of time?; (b) would such low genetic, karyotype, and morphological variability be related to the low number of extant crocodilian species?

Crocodylomorpha (a clade that comprises living and extinct crocodilians) first appeared roughly 250 million years ago, and its 28 existing species are among the biggest living ectothermic animals. As a result, their survival over such a long geological period is of great evolutionary importance. They do, however, have a rich fossil history that includes hundreds of extinct species, revealing a hidden past of incredible variety and complexity [95, 96]. Oaks [65] has questioned the traditional notion of crocodiles as old “living fossils,” arguing that most extant crocodilians are remnants of formerly successful lineages in terms of diversity and range. Crocodylomorpha is the only pseudosuchians to have survived the Triassic-Jurassic (TJ) extinction event, which happened around 200 million years ago [97, 98]. Furthermore, after the mid-Miocene climatic optimum, there was a huge drop in crocodilian diversity, which coincided with global cooling and glacial advancement. During the Pliocene, the number of taxa is believed to have decreased from around 26 to 8, representing the greatest extinction rate over the previous 100 million years [99]. As a result, the selection of an “evolutionary package” with similar genomic, chromosomal, morphology, and physiology to what is currently observed among extant species most likely resulted from drastic demographic declines or founder events and represented evolutionary responses to a long-term bottleneck history.

## Conclusions

This study is the first to offer a comparative mapping of the satDNA families in Alligatoridae. We observe some level of interspecific divergence even with so strong sequence conservatism through Caimaninae. With the results, we learn that satDNA orthologs indicate their evolutionary process according to different hybridization patterns. After rounds of mining, we discover the alligators’ satellitomes are one of the smallest satDNA libraries described so far, with just four groups of satDNAs and four sequences species-specific between all species, possibly showing as ancestral features for the group, conserved throughout the crocodilians for a long time. With additional studies about repetitive DNAs in the other families of Crocodylia, it is important to demonstrate the evolution of these sequences and provide more information about the chromosomal evolution in reptiles.

## Methods

### Samples, DNA extraction, and chromosomal preparation

Table 2 summarizes the collecting sites, number, and sex of individuals used in this investigation. The sampling is similar to that previously examined by [66]. In vitro blood cultures were used to obtain chromosomal preparations [100, 101]. The usual phenol–chloroform-isoamyl alcohol procedure was used to extract genomic DNA (gDNA) from blood stored in 100% ethanol [102].

**Table 2:** Species, sample size (*N*), sex, and locality of the analyzed individuals. The species whose satellitomes were studied are highlighted in bold

Species	*N*	Locality/origin of samples	
*Caiman crocodilus* (Spectacled caiman)	2♀, 2♂	Amazonas (BR) (Amazon Basin)	3° 22′ 34.7″ S 60° 19′ 20.7″ W
***Caiman latirostris* (Broad-snouted caiman)**	4♀, 6♂	São Paulo (BR) (Cerrado)	22° 33′ 53.1″ S 48° 00′ 35.2″ W
***Caiman yacare* (Yacare caiman)**	2♀, 8♂	Mato Grosso (BR) (Pantanal)	16° 19′ 32.0″ S 57° 46′ 35.7″ W
***Melanosuchus niger* (Black caiman)**	2♀, 2♂	Amazonas (BR) (Amazon Basin)	3° 25′ 50.4″ S 66° 02′ 35.0″ W
*Paleosuchus palpebrosus* (Cuvier’s dwarf caiman)	3♀, 3♂	Pará (BR) (Amazon Basin)	1° 18′ 19.7″ S 48° 19′ 05.0″ W
***Paleosuchus trigonatus* (Schneider’s smooth-fronted caiman)**	3♀, 4♂	Amazonas (BR) (Amazon Basin)	3° 06′ 52.0″ S 60° 01′ 58.0″ W
*Alligator mississippiensis* (American alligator)	2♀, 2♂	Canberra University collection (Australia)	
***Alligator sinensis* (Chinese alligator)**	4♀, 2♂	Private collections (Germany)	

### Sequencing data

Two broad-snouted caimans *C. latirostris* and the Schneider’s smooth-fronted caiman *P. trigonatus* were selected for low-pass shotgun sequencing on the BGISEQ-500 platform at BGI (BGI Shenzhen Corporation, Shenzhen, China), yielding 2.76, 2.76, and 2.67 Gb, respectively (Additional file 2: Table S7). Raw reads are available in the Sequence Read Archive from the NCBI (SRA-NCBI) under the accession numbers: SRR19901397 (*C. latirostris* male), SRR19901398 (*C. latirostris* female), SRR19901554 (*P. trigonatus* female)*.* To search and compare satDNAs in other Alligatoridae species, we also collected genomic data available in the SRA-NCBI for the Yacare caiman *Caiman yacare* (SRR1609243), the black caiman *Melanosuchus niger* (SRR1609245) and for the Chinese alligator *Alligator sinensis* (SRR953089), thus encompassing all the extant Alligatoridae genera. The general features of sequencing data are summarized in Additional file 2: Table S8.

### Satellite DNA characterization and comparative analyses

After gathering sequencing data for all the species as mentioned earlier, we performed a quality (Q > 30) and adapter trimming with Trimmomatic [103] for each library separately. After that, we proceeded to the characterization of satDNAs in each species. We performed several iterations of RepeatExplorer2 [36] and filtered the identified satDNAs with DeconSeq [104] following the protocol of [6]. We analyzed 2 × 500,000 reads in each iteration until no low- or high-confidence satellite DNA was found. After multiple iterations, we filtered and removed multigene families (5S rDNA and/or U snDNA) from the catalog. Then, we performed a similarity search among the remaining sequences with RepeatMasker using a custom python script (https://github.com/fjruizruano/ngs-protocols/blob/master/rm_homology.py), grouping them as the same sequence variant (≥ 95% of similarity), variant (≥ 80% of similarity) or different satDNA sharing a same superfamily (≥ 50% of similarity) in each species [6]. After that, we estimated Kimura’s divergence, using Kimura 2-parameter model from the script calcDivergenceFromAlign.pl of RepeatMasker suite and abundance values for all satDNAs families with the “cross_match” option in RepeatMasker software [105], using 2 × 5,000,000 reads for each library, except for *Melanosuchus niger* and *Caiman yacare*, because their libraries had fewer reads, performing the analysis with 2 × 1,213,376 and 2 × 1,608,245, respectively (Table 1; Additional file 1: Fig. S1). Genomic abundance of each satDNA was given as the number of mapped reads in each satDNA divided by the number of analyzed nucleotides. Finally, we classified each satellite based on decreasing abundance order, as Ruiz-Ruano et al. [6] suggested. The specific features of each satDNA are observed in Table 1. Each catalog of satDNAs was deposited on the GenBank with accession numbers OP169024–OP169026 (*A. sinensis*), OP169027–OP169032 (*C. yacare*), OP169033–OP169038 (*M. niger*), OP169039–OP169049 (*P. trigonatus*), and OP169050–OP169062 (*C. latirostris*). One additional and independent RepeatExplorer2 run was performed with a concatenated genomic library containing 150,000 reads from each species, using the “Perform comparative analysis” option.

To compare the satellitomes of multiple species, we performed a similarity search with RepeatMasker (https://github.com/fjruizruano/ngs-protocols/blob/master/rm_homology.py) considering all the de novo-characterized satDNA sequences. Then, we aligned the monomers of all satDNAs showing at least 50% similarity with MUSCLE [106]. In addition, we generated individual self-dotplots of the satDNA sequences and a general one with Flexidot [107].

For a general visualization of abundance and presence/absence of each satDNA in the different species, we ran RepeatMasker [105] against the complete catalog of Alligatoridae using each of the genomic libraries. After that, we normalized read coverage of the samples relative to single-copy genes. For this, we retrieved three single-copy genes in Sauropsida (options: Present in all species; Single-copy in all species) in the OrthoDB (https://www.orthodb.org/; accessed in July 30th) and mapped the genomic libraries against the genes using bowtie2 [108] with the preset values –sensitive and –local. Then, a normalization factor was calculated as: [(number of mapped reads x read sizes x gene sizes)/number of analyzed reads] (Additional file 2: Table S9). A final step of summing up the log10 of normalized read counts from RepeatMasker (0 to 20% of Kimura divergence) was performed. With the final matrix (Additional file 2: Table S10), we generated a Clustermap (Fig. 1) with Seaborn using the seaborn.clustermap function (https://seaborn.pydata.org/generated/seaborn.clustermap.html).

Taking advantage of the fact that the genome of *Alligator sinensis* is available in the NCBI (GCA 000455745.1), we also conducted a BLAST (blastn, word size = 11, e-value = 1e-6) to search the entire list of satDNAs against this genome that was assembled using Illumina Hiseq2000 [67]. We did not perform any structural or quantitative analysis on array sizes and/or organization because only short reads were employed for this assembly [67]. As a result, BLAST searches provided more useful information on the presence or absence of satDNAs in the genome of *A. sinensis*. To get an estimation of the degree of tandem structure for the satDNAs in this species, we calculated the Tandem Structure Index (TSI), as demonstrated in [71]. This value is calculated as the quotient of the number of paired reads mapped against a satDNA and the total number of reads (https://github.com/fjruizruano/SatIntExt). Thus, higher TSI values indicate the occurrence of longer arrays in the analyzed species. One must note that once the FISH probes were labeled and hybridized in groups, the TSI values are not completely suitable for comparison with the FISH results of satDNAs within groups 1 to 4.

### Primer design and polymerase chain reaction (PCR)

We designed primer pairs for 12 satellite DNA families characterized from *C. latirostris* and two satellite DNA families characterized for *A. sinensis*, creating convergent primers for satellites larger than 1000 bp and divergent primers for satellites smaller than 1000 bp. We verified if those primer pairs anchors in conserved regions of monomers and used them to PCR-amplify in all Alligatoridae species. The PCRs contained 1 × PCR buffer, 1.5 mM of MgCl_2_, 200 µM of each dNTP, 0.5 µL of each primer, 10–100 ng/µL of gDNA, and 0.2 µl of Taq DNA polymerase in a total volume of 25 µL. The PCR program included an initial denaturation at 95 °C for 7 min, followed by 34 cycles at 95 °C for 45 s, 61 °C for 1 min, 72 °C for 1 min, and a final extension at 72 °C for 7 min. The PCR products were checked in 2% agarose gel.

### Probe labeling and fluorescence in situ hybridization (FISH)

Except for ClaSat03-183, ClaSat09-285, ClaSat12-24, and AsiSat03-96, all the other satDNAs were successfully amplified and the PCR products were labeled with Atto550-dUTP (red) or Atto488-dUTP (green) according to the manufacturer’s recommendations using the Nick-Translation mix kit (Jena Bioscience, Jena, Germany). The probes were then hybridized in all other Alligatoridae species according to the methodology reported by [109]. To corroborate the FISH results, at least 30 metaphase spreads were examined in each individual. Photos were obtained with CoolSNAP on an Olympus BX50 microscope (Olympus Corporation, Ishikawa, Japan), and the images were processed using Image-Pro Plus 4.1 software (Media Cybernetics, Silver Spring, MD, USA).

### Supplementary Information

**Additional file 1: Figures S1-S6. Fig S1—**All-against-all dotplot of Caimaninae satellite DNA, with the division of these sequences among the three groups presented in the analysis in green (group 1), red (group 2), and blue (group 3), and the similarity among each satDNA family, represented by a white to the black color ladder. **Fig S2—**This figure shows the alignment between two consecutive repeats and the next two. (a) The scheme shows the four 41 bp repeats (A, B, C and D) aligned two by two with their corresponding 21/20 bp subunits (alpha and beta) distinguished by being shaded in gray (alpha) or blue (beta). Asterisks indicate similarity between aligned sequences. Nucleotide positions that are divergent between alpha and beta subunits of each repeat unit have been marked in red. (b) The alignment of the four 41 bp repetitive units (A, B, C and D) is shown on one side and the separate alignments of the alpha (gray) and beta (blue) subunits are shown on the other. Asterisks indicate similarity between aligned sequences. Divergent nucleotides are not shaded. (c) Multiple alignment of all alpha and beta subunits. Asterisks indicate similarity between aligned sequences. Divergent nucleotides are not shaded. Consistent with Table S2, it can be observed in Figure S2 that inter-repeat alignments show more conserved nucleotide positions than intra-repeat ones. In addition, it can be observed that the most divergent part between alpha and beta subunits occurs at the 3' end. **Fig S3—**The figure is an example of the internal organization of long satellites. In this case, ClaSat06-1063. As can be seen, the repetitive unit of this satellite is made up of 12 subrepeats of 41 bp between which the average divergence is 0.30, followed by a short intervening sequence of 23 bp and then 6 repeats of a sequence of 81 bp (41 + 40 bp) with an average divergence between subrepeats of 0.35. Finally, a 62 bp fraction of the latter subrepeats. Therefore, the repetitive unit of this satellite has evolved through different cycles of duplication and divergence first from 40 bp subunits (but not 20 bp) and then from 80 bp subunits through a complex process in which partial sequences of the 40 bp subunit have been interspersed (pointing to unequal crossing over as a molecular tool towards the consolidation of a current 1063 bp unit). In red, the average sequence of 41 bp subunits and 81 bp subunits. **Fig S4—**Phylogenetic tree (minimum spanning tree, mst) comparing alpha (red) and beta (blue) subunits of the ClaSat05-40 satellite. We can observe two distinct clades, one formed by alpha sequences and others by beta sequences. **Fig S5—**Metaphase chromosomes from C. crocodilus (a–d), C. latirostris (e–h) and C. yacare (i–l) after in situ mapping with satDNA probes belonging to group 1 (ClaSat06-1063; ClaSat07-320; ClaSat08-800 and ClaSat11-547). The satDNA FISH signals are highlighted in green (ATTO488 labeled) or red (ATTO550 labeled) and the chromosomes were counterstained with DAPI (blue). Scale bar = 20 μm.** Fig S6—**Metaphase chromosomes from M. niger (a–d), P. palpebrosus (e–h) and P. trigonatus (i–l) after in situ mapping with satDNA probes belonging to group 1 (ClaSat06-1063; ClaSat07-320; ClaSat08-800 and ClaSat11-547). The satDNA FISH signals are highlighted in green (ATTO488 labeled) or red (ATTO550 labeled) and the chromosomes were counterstained with DAPI (blue). Scale bar = 20 μm. 

**Additional file 2: Tables S1-S10. Table S1—**Within-group pairwise similarity of satDNAs.** Table S2—**Summary of the results obtained from the read clustering analysis with multiple species analyzed simultaneously.** Table S3—**We have compared hundreds of repetitive units from each satellite and compared the mean divergence that exists between them (inter-repeat divergence) with the mean divergence that exists between the subunits that compose each repetitive unit (intra-repeat divergence). For the case of the sequences of the Group 1 constituted by repetitive units of 41 bp, we have considered each of its subunits (alpha and beta) of 21 and 20 bp, respectively, obtaining that the intra-repeat divergence was always greater than the inter-repeat divergence.** Table S4—**As for the Group 1 satellites, we have analyzed the 60 bp long satellites of Group 2. Here we verified that their repetitive units were made up of subunits of 20 bp each, which we have named alpha, beta, and gamma. The table shows the analysis of intra-repeats versus inter-repeats divergences, and it can also be seen here that the former is greater than the latter. **Table S5—**While Groups 1 and 2 were more homogeneous, Group 3 sequences are characterized as heterogeneous ones, as their sequences were derived from a repetition of two subunits of 29 bp and 11 bp. This table demonstrates that the divergence between alpha (29 bp) and beta (11 bp) subrepeats is greater than the divergence between complete repeats of these satellites. Thus, looking at their structure, we can affirm that the Group 3 satellites are not formed by repetitive units of 20 bp. In any case, it is possible that the ancestral satellite that gave rise to the group 3 satellites had a length of 29 bp and that later rearrangements, possibly mediated by unequal crossing over (see, for example, Navajas-Pérez et al. 2005 for RAYSI satDNA), generated a 40 bp repetitive unit formed by one unit plus one third of another, being now the unit of homogenization of these satellites.** Table S6—**Summary of the BLAST searches against Alligator sinensis genome.** Table S7—**Main features of the analyzed genomic libraries.** Table S8—**Results of RepeatMasking against the Repbase database.** Table S9—**Read mapping against single-copy genes for normalization of satDNA abundance.** Table S10—**log10 of normalized abundances of each satDNA, considering RepeatMasker counts from 0 to 20% of divergence.

## Data Availability

The datasets generated during and/or analyzed during the current study are available in the NCBI database (https://www.ncbi.nlm.nih.gov/bioproject/) under accession numbers OP169024–OP169026 (*A. sinensis*), OP169027–OP169032 (*C. yacare*), OP169033–OP169038 (*M. niger*), OP169039–OP169049 (*P. trigonatus*), and OP169050–OP169062 (*C. latirostris*) and, in the SRA-NCBI for the Yacare caiman *Caiman yacare* (SRR1609243), the black caiman *Melanosuchus niger* (SRR1609245), and for the Chinese alligator *Alligator sinensis* (SRR953089). All other data generated or analyzed during this study are included in this published article and its supplementary information files.
