# Supplementary material for: Evolution of ancient satellite DNAs in extant alligators and caimans (Crocodylia, Reptilia)
Source: BMC Biol. 2024 Feb 27;22:47. doi: 10.1186/s12915-024-01847-8 (PMC10900743; doi:10.1186/s12915-024-01847-8)
Supplement: Supplementary file 1 — Additional file 1: Figures S1-S6. Fig S1—All-against-all dotplot of Caimaninae satellite DNA, with the division of these sequences among the three groups presented in the analysis in green (group 1), red (group 2), and blue (group 3), and the similarity among each satDNA family, represented by a white to the black color ladder. Fig S2—This figure shows the alignment between two consecutive repeats and the next two. (a) The scheme shows the four 41 bp repeats (A, B, C and D) aligned two by two with their corresponding 21/20 bp subunits (alpha and beta) distinguished by being shaded in gray (alpha) or blue (beta). Asterisks indicate similarity between aligned sequences. Nucleotide positions that are divergent between alpha and beta subunits of each repeat unit have been marked in red. (b) The alignment of the four 41 bp repetitive units (A, B, C and D) is shown on one side and the separate alignments of the alpha (gray) and beta (blue) subunits are shown on the other. Asterisks indicate similarity between aligned sequences. Divergent nucleotides are not shaded. (c) Multiple alignment of all alpha and beta subunits. Asterisks indicate similarity between aligned sequences. Divergent nucleotides are not shaded. Consistent with Table S2, it can be observed in Figure S2 that inter-repeat alignments show more conserved nucleotide positions than intra-repeat ones. In addition, it can be observed that the most divergent part between alpha and beta subunits occurs at the 3' end. Fig S3—The figure is an example of the internal organization of long satellites. In this case, ClaSat06-1063. As can be seen, the repetitive unit of this satellite is made up of 12 subrepeats of 41 bp between which the average divergence is 0.30, followed by a short intervening sequence of 23 bp and then 6 repeats of a sequence of 81 bp (41 + 40 bp) with an average divergence between subrepeats of 0.35. Finally, a 62 bp fraction of the latter subrepeats. Therefore, the repetitive unit of this satellite [file 12915_2024_1847_MOESM1_ESM.docx]

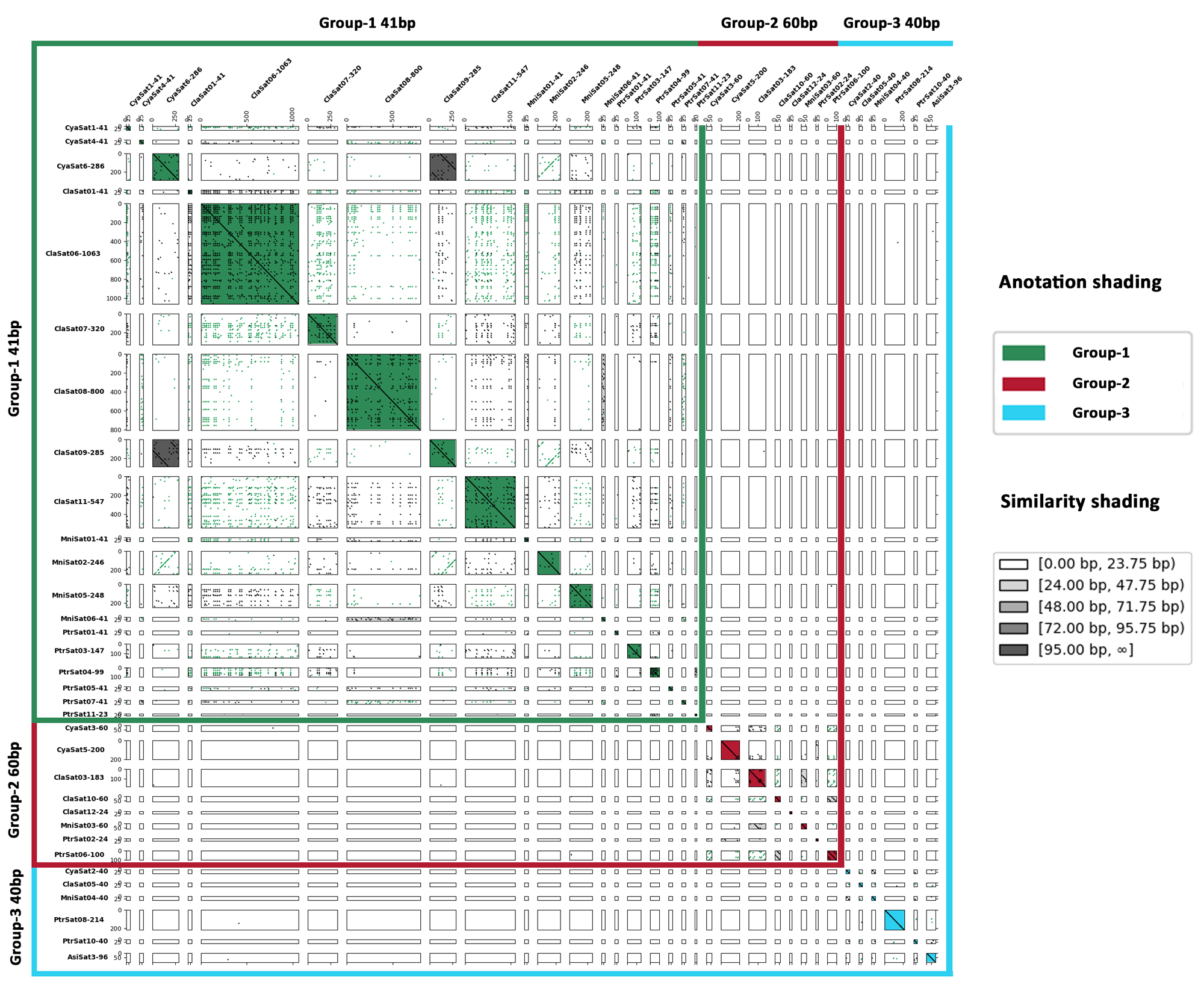


**Figure S1.** All-against-all dotplot of Caimaninae satellite DNA, with the division of these sequences among the three groups presented in the analysis in green (group 1), red (group 2), and blue (group 3), and the similarity among each satDNA family, represented by a white to the black color ladder.


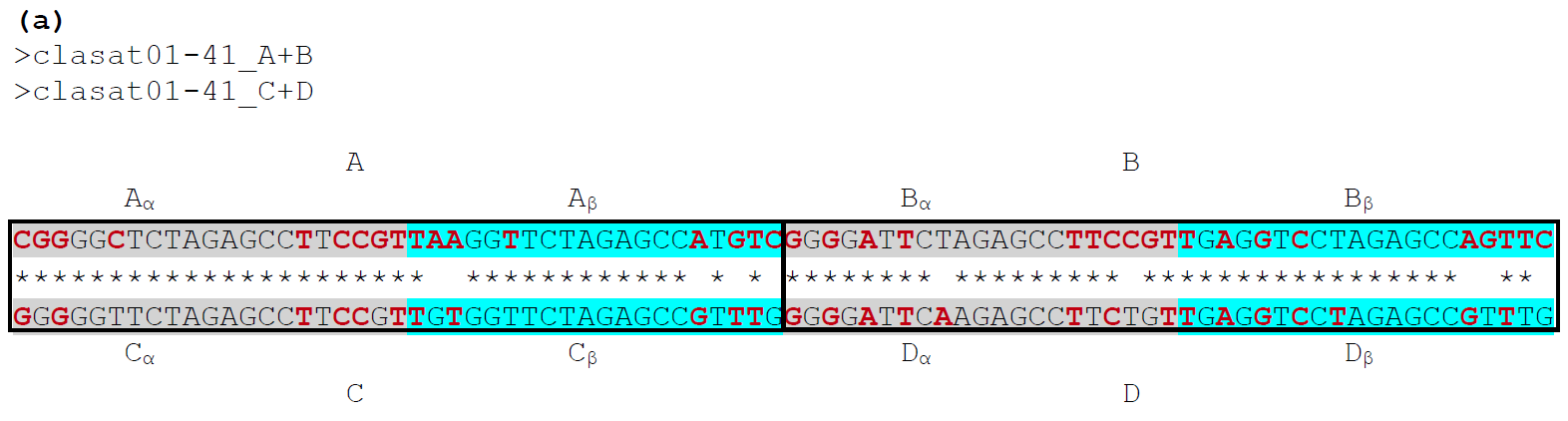


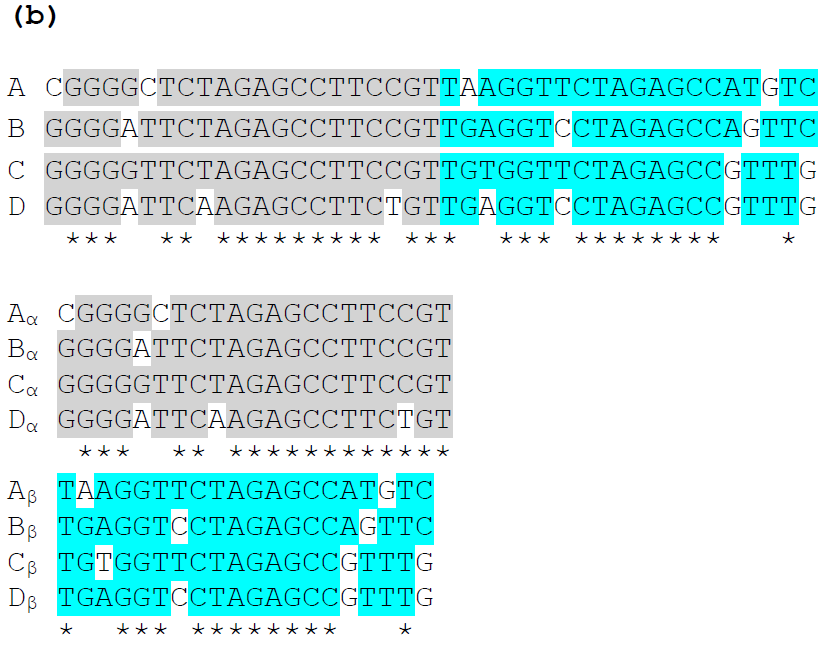

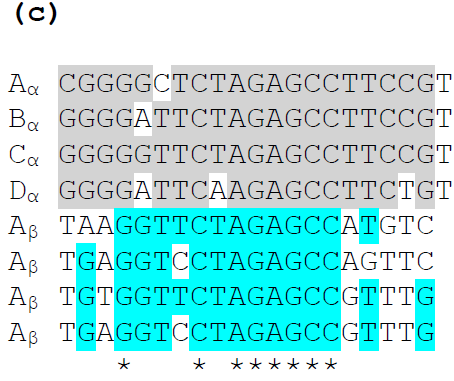


**Figure S2.** This figure shows the alignment between two consecutive repeats and the next two. **(a)** The scheme shows the four 41 bp repeats (A, B, C and D) aligned two by two with their corresponding 21/20 bp subunits (alpha and beta) distinguished by being shaded in gray (alpha) or blue (beta). Asterisks indicate similarity between aligned sequences. Nucleotide positions that are divergent between alpha and beta subunits of each repeat unit have been marked in red. **(b)** The alignment of the four 41 bp repetitive units (A, B, C and D) is shown on one side and the separate alignments of the alpha (gray) and beta (blue) subunits are shown on the other. Asterisks indicate similarity between aligned sequences. Divergent nucleotides are not shaded. **(c)** Multiple alignment of all alpha and beta subunits. Asterisks indicate similarity between aligned sequences. Divergent nucleotides are not shaded. Consistent with Table S2, it can be observed in Figure S2 that inter-repeat alignments show more conserved nucleotide positions than intra-repeat ones. In addition, it can be observed that the most divergent part between alpha and beta subunits occurs at the 3' end.


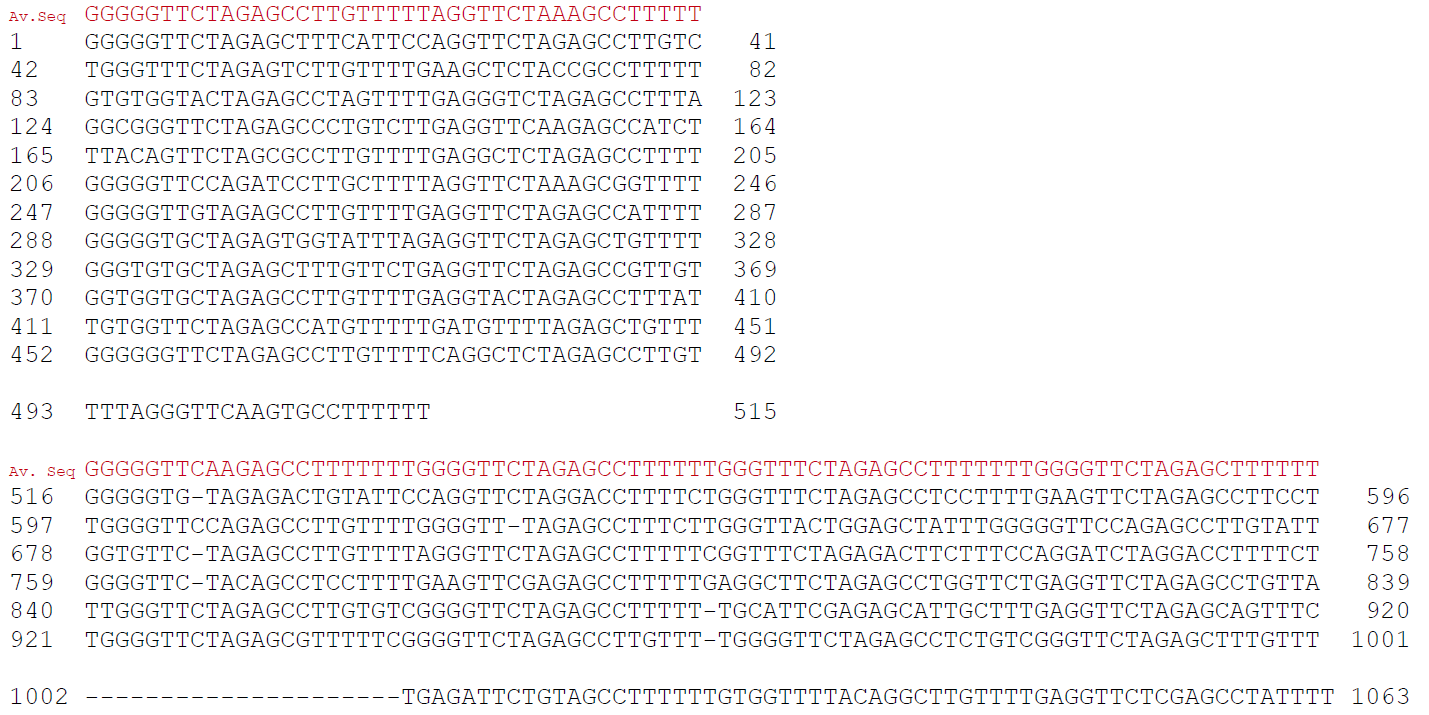


**Figure S3.** The figure is an example of the internal organization of long satellites. In this case, ClaSat06-1063. As can be seen, the repetitive unit of this satellite is made up of 12 subrepeats of 41 bp between which the average divergence is 0.30, followed by a short intervening sequence of 23 bp and then 6 repeats of a sequence of 81 bp (41+40 bp) with an average divergence between subrepeats of 0.35. Finally, a 62 bp fraction of the latter subrepeats. Therefore, the repetitive unit of this satellite has evolved through different cycles of duplication and divergence first from 40 bp subunits (but not 20 bp) and then from 80 bp subunits through a complex process in which partial sequences of the 40 bp subunit have been interspersed (pointing to unequal crossing over as a molecular tool towards the consolidation of a current 1063 bp unit). In red, the average sequence of 41 bp subunits and 81 bp subunits.


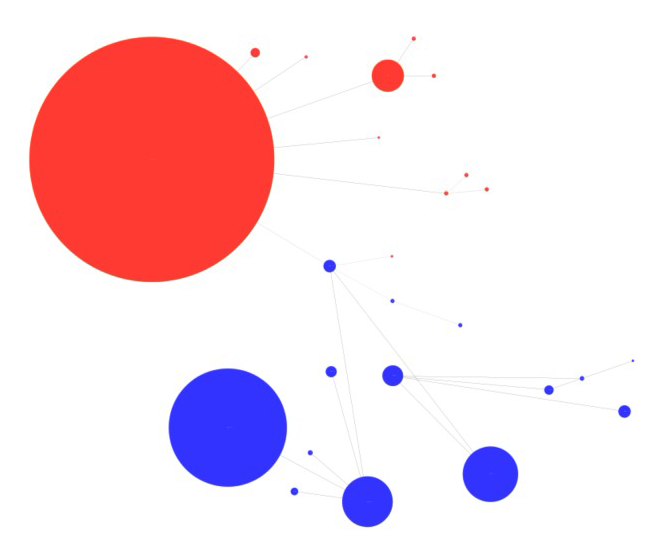


**Figure S4**. Phylogenetic tree (minimum spanning tree, mst) comparing alpha (red) and beta (blue) subunits of the ClaSat05-40 satellite. We can observe two distinct clades, one formed by alpha sequences and others by beta sequences.


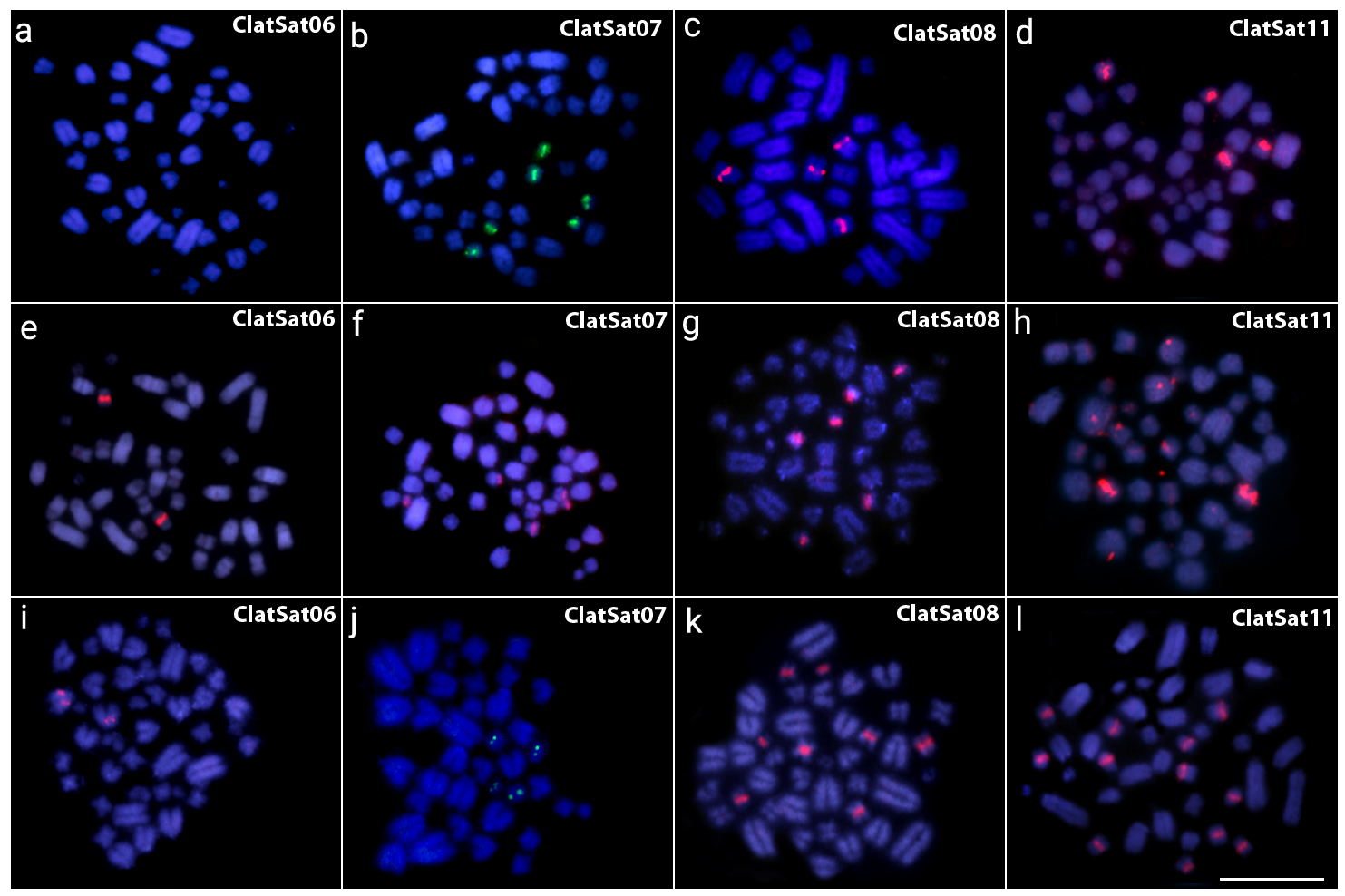


**Figure S5.** Metaphase chromosomes from *C. crocodilus* (a–d), *C. latirostris* (e–h) and *C. yacare* (i–l) after in situ mapping with satDNA probes belonging to group 1 (ClaSat06-1063; ClaSat07-320; ClaSat08-800 and ClaSat11-547). The satDNA FISH signals are highlighted in green (ATTO488 labeled) or red (ATTO550 labeled) and the chromosomes were counterstained with DAPI (blue). Scale bar = 20μm


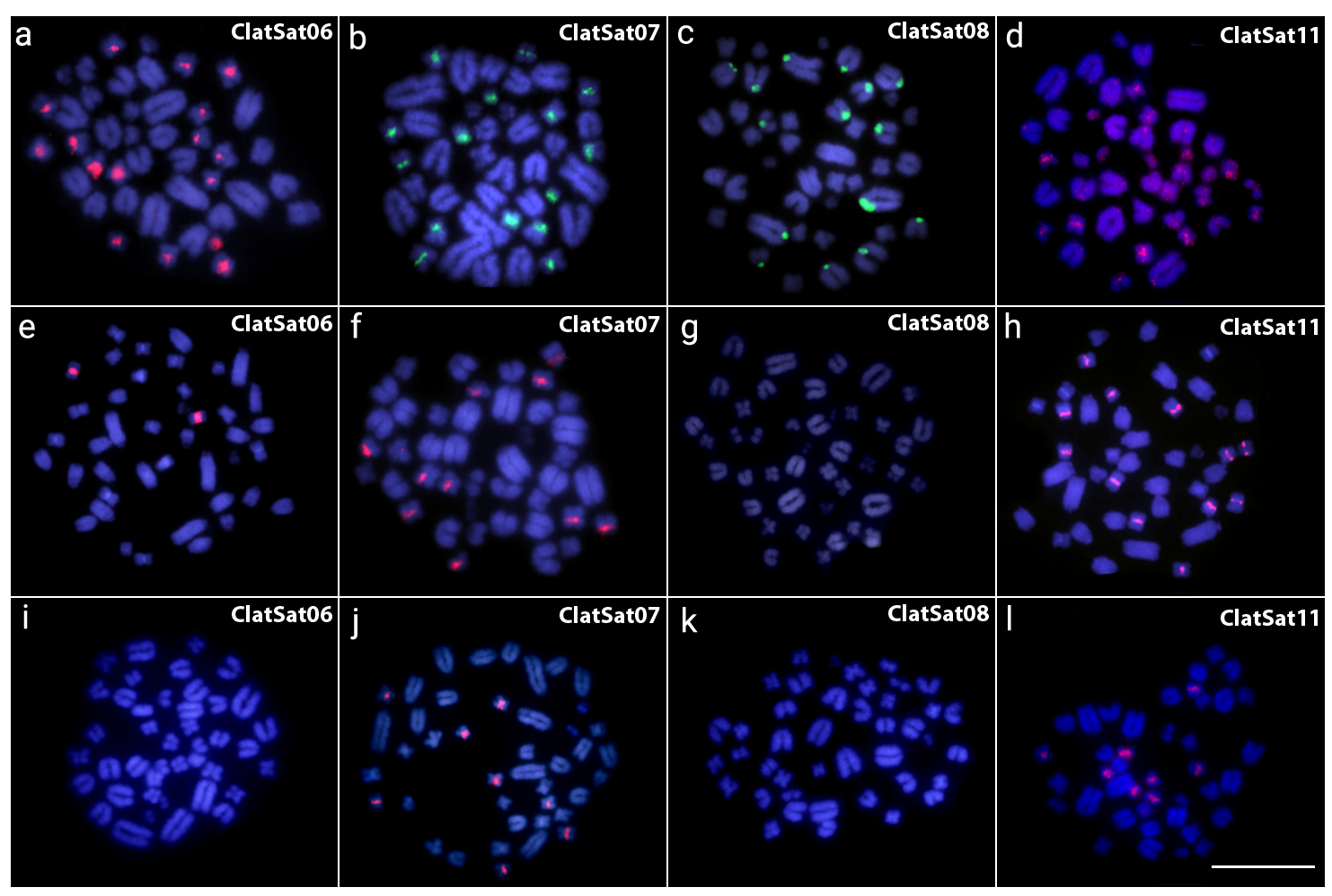


**Figure S6.** Metaphase chromosomes from *M. niger* (a–d), *P. palpebrosus* (e–h) and *P. trigonatus* (i–l) after in situ mapping with satDNA probes belonging to group 1 (ClaSat06-1063; ClaSat07-320; ClaSat08-800 and ClaSat11-547). The satDNA FISH signals are highlighted in green (ATTO488 labeled) or red (ATTO550 labeled) and the chromosomes were counterstained with DAPI (blue). Scale bar=20μm
